# Hepatoprotective and nephroprotective effects of *Tessaria integrifolia* Ruiz and Pav. on diclofenac-induced toxicity in rats

**DOI:** 10.14202/vetworld.2023.1933-1939

**Published:** 2023-09-23

**Authors:** Paquito D. Mendoza-Fernández, Carmen R. Silva-Correa, Víctor E. Villarreal-La Torre, Cinthya L. Aspajo-Villalaz, Abhel A. Calderón-Peña, Jorge Del Rosario-Chávarri

**Affiliations:** 1 Department of Pharmacy and Biochemistry, Escuela de Posgrado, Universidad Nacional de Trujillo, Perú; 2 Department of Pharmacology, Facultad de Farmacia y Bioquímica, Universidad Nacional de Trujillo, Perú; 3 Department of Biological Chemistry and Animal Physiology, Facultad de Ciencias Biológicas, Universidad Nacional de Trujillo, Perú

**Keywords:** diclofenac, hepatoprotective, nephroprotective, *Tessaria integrifolia*

## Abstract

**Background and Aim:**

*Tessaria integrifolia* Ruiz and Pav. (also known as “Pájaro bobo”) is known for its medicinal properties, including antiulcer, antiasthmatic, leishmanicidal, antipyretic, antispasmodic, diuretic, anti-inflammatory, analgesic, and hepatoprotective effects. Therefore, we aimed to evaluate its hepatoprotective and nephroprotective effects using a rat model of diclofenac-induced toxicity.

**Materials and Methods:**

We administered three different doses of the methanolic extract of *T. integrifolia* (100, 200, and 400 mg/kg/day orally) and compared them with the commercial medicine silymarin (100 mg/kg orally). The rats received the *T. integrifolia* extracts for 5 days, and on days 3 and 4, 1 h after receiving the extracts, diclofenac was administered intraperitoneally at a dose of 50 mg/kg. The animals were euthanized 48 h after the last diclofenac injection, and blood samples were obtained to measure biochemical parameters related to liver and kidney function, such as alanine aminotransferase (ALT), aspartate aminotransferase (AST), bilirubin, cholesterol, triglycerides, creatinine, and urea. Kidney and liver tissues were preserved in 10% formaldehyde and sent for histopathological analysis.

**Results:**

The results show that *T. integrifolia* has hepatoprotective and nephroprotective effects. These effects are verified by the lower blood levels of ALT, AST, urea, and creatinine compared to the diclofenac group, which exhibited elevated biochemical parameters. In addition, histopathological analysis showed that the groups that received *T. integrifolia* did not display necrosis or infiltration, which were observed in the diclofenac group.

**Conclusion:**

The methanolic extract of *T. integrifolia* has hepatoprotective and nephroprotective effects, with the highest protective activity observed at a dose of 400 mg/kg/day.

## Introduction

Nonsteroidal anti-inflammatory drugs (NSAIDs) are among the most extensively consumed drugs worldwide. They are widely used in treating pain and processes induced by mild-to-moderate inflammation [1], exerting anti-inflammatory, analgesic, and antipyretic effects by suppressing prostaglandin (PG) synthesis, which inhibits the cyclooxygenase (COX) enzyme [2]. Nonselective NSAIDs inhibit COX-1 and COX-2. While COX-1 is a constitutive enzyme found in the kidneys, COX-2 is an inducible enzyme that increases in tissues in response to injury and inflammation [3].

Diclofenac, an NSAID extensively used in the treatment of rheumatoid arthritis, has therapeutic benefits but also induces side effects related to the liver and kidneys [4–6]. It affects various organs, including the lungs, kidneys, liver, stomach, and heart [2]. Moreover, it causes mitochondrial damage by generating reactive oxygen species (ROS) and inhibiting enzymatic and nonenzymatic activity in liver and kidney tissues [7–9]. Nephrotoxicity and hepatotoxicity are severe health disorders caused by drug consumption, including NSAIDs, necessitating the investigation of protective compounds to prevent this damage. Thus, research on medicinal plants has increased in recent years, as they are a valuable resource of protective compounds and are used to develop safer and more potent phytochemical drugs [10, 11].

*Tessaria integrifolia*, or “Pájaro bobo,” belongs to the *Asteraceae* family. This shrub, reaching 3–10 m high, has lanceolate leaves, secondary flowers, andterminal inflorescences with yellow capitula. It is commonly found in vegetation along rivers in Peru’s coastal and jungle regions [12]. Known for its medicinal properties, including antiulcer, antiasthmatic, leishmanicidal, antipyretic, antispasmodic, diuretic, and anti-inflammatory effects, this plant species has been used to induce analgesia when treating liver and kidney stones [13]. Chemical compounds, such as caffeoylquinic acid, eudesmane derivatives, flavones, sesquiterpenes, lignans, α-terthienyl, bisthenyl derivatives, β-amyrin acetate, and squalene, have been found in the roots, stems, leaves, and flowers of *T. integrifolia* [14, 15]. Notably, the methanolic extract of *T. integrifolia* leaves contains phenolic compounds, eudesmanes, caffeolic acid, lignans, sesquiterpenes, and flavonoids [16]. Furthermore, a eudesmane-type compound with leishmanicidal activity has been reported in the fluid extract of its leaves [13].

The search for therapeutic alternatives has been increasing given the adverse effects associated with consuming drugs such as NSAIDs, which cause problems of hepatotoxicity and nephrotoxicity despite their prevalence. Natural medicine, which involves the use of medicinal plants with protective effects against the toxicity induced by conventional drugs, has emerged as a potential solution. Therefore, we evaluated the hepatoprotective and nephroprotective effects of *T. integrifolia* in a rat model of diclofenac-induced toxicity.

## Materials and Methods

### Ethical approval

The study was approved by the Ethics Committee of the Faculty of Pharmacy and Biochemistry of Universidad Nacional de Trujillo with the document COD. N°: BLL002-2022/CEIBYF.

### Study period and location

The study was conducted from January to July 2022. All processes were performed in Toxicology Laboratory, School of Pharmacy and Biochemistry, Universidad Nacional de Trujillo.

### Biological material

Three-month-old male and female Holtzman albino rats (250–300 g) were utilized for this study. They were acquired from the National Institute of Health and kept in the animal laboratory at the School of Pharmacy and Biochemistry of the Universidad Nacional de Trujillo. All rats were housed in metal cages, and sterile shavings were used as bedding material. The animals were conditioned in an environment with a controlled temperature of 25°C ± 2°C and a 12-h light–dark cycle, and they were provided with standard commercial rat food and water *ad libitum*.

### Vegetal material

Leaves of *T. integrifolia* (5 kg) were collected from the Chamán River in the Chepén Province of the La Libertad Region at an altitude of 135 m.a.s.l., with latitude 07°13’36’’ and longitude 79°25’45’’. A specimen of the plant was taken to the Herbarium Truxillense (HUT) of the National University of Trujillo for botanical classification and assigned the code HUT N° 59577.

### Preparation of the extract

The leaves were washed with distilled water and air-dried. Mechanical milling was used to pulverize the leaves into a fine powder, which was then stored in an amber glass container. Subsequently, 25 g of the powder was macerated with 250 mL of methanol (J. T. Baker, Spain) at 22°C for 7 d with occasional shaking. The macerate was sterile-filtered in a biosafety cabinet (FCL 180-C4; Colombia) and subjected to rotary evaporation (Heidolph®, Germany) at 40°C and variable pressure, starting at 350 mbar and gradually reducing to 80 mbar. The resulting material was placed in an oven at 40°C until a dry extract was obtained, which was stored in a freezer (Biobase, China) at −20°C. The dry extract was dissolved in water at different concentrations before being administered at 100, 200, and 400 mg/kg/day [17].

### Determination of extraction yield

The percentage of extraction yield (EY%) was calculated as described by Truong *et al*. [18]:


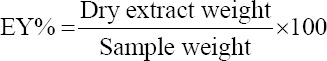


#### Evaluation of hepatoprotective and nephroprotective activity

The rats were divided into six experimental groups comprising five specimens each. They were fed through orogastric gavage using a flexible plastic feeding tube. Group I (negative control) received 1 mL of sterile water daily for 5 days. Group II (positive control: diclofenac) received diclofenac (50 mg/kg intraperitoneal) on days 3 and 4. Groups III, IV, and V were orally administered *T. integrifolia* extract at doses of 100, 200, and 400 mg/kg/day, respectively, for 5 d, along with a single 50 mg/kg dose of diclofenac sodium (Sigma-Aldrich, USA) intraperitoneally 1 h after extract administration on days 3 and 4. Group VI (standard: silymarin) received the standard medication, silymarin (Sigma-Aldrich), orally at 100 mg/kg/day for 5 days, with diclofenac (50 mg/kg intraperitoneal) administered 1 h after silymarin administration on days 3 and 4 [17].

#### Analysis of biochemical parameters

Blood was collected from the right ventricle of the heart. The obtained serum was used to determine the levels of biochemical parameters, such as aspartate aminotransferase (AST), alanine aminotransferase (ALT), bilirubin, cholesterol, triglycerides, urea, and creatinine, using UV-spectrophotometric methods (Orion AquaMate 8000 – Thermo Scientific; USA) [17].

### Histopathological study

The animals were euthanized 48 h after the last diclofenac injection under anesthesia with sodium pentobarbital (60 mg/kg intraperitoneal). The liver and kidneys of each animal were extracted and placed in a 10% formaldehyde solution (Spectrum Chemical MFG Corp., USA) for 8 days. Subsequently, they were dehydrated, embedded in paraffin, sectioned at 5 mm, and stained with hematoxylin/eosin [19].

### Statistical analysis

The data obtained were analyzed using the R software (Version 4.1.1) for Windows® (https://www.npackd.org/p/r/4.1.1). The data were subjected to an analysis of variance, followed by the Tukey test for *post hoc* comparisons. Statistical significance was considered at p < 0.05.

## Results

### Extraction yield

Table-1 presents the color, consistency, odor, extract weight, and percentage yield of the methanolic extract derived from *T. integrifolia* leaves. The extract exhibited a dark blackish-green color, a semi-solid consistency, and an odor. Notably, the dry extract weighed 9.77 g, and the percentage yield was 39.09% (w/w).

**Table-1:** Percentage yield and physical characteristics of methanolic extract of *Tessaria integrifolia* leaves.

Physical parameters	Result
Color	Dark-blackish-green
Odor	Aromatic
Consistency	Semi-solid
Dry extract weight (g)	9.77
Yield (%)	39.09

### Analysis of biochemical parameters

**Figure-1:**
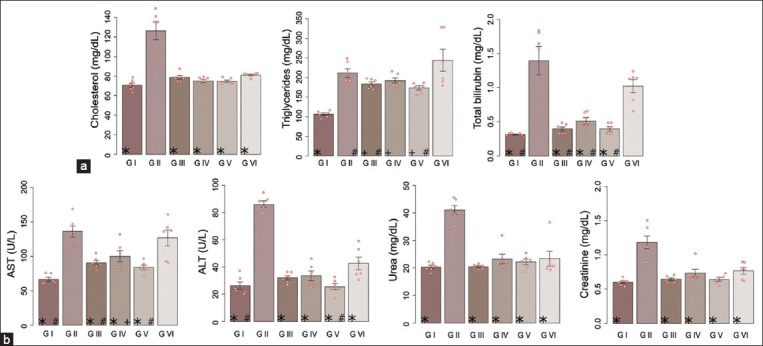
Levels of biochemical parameters in the experimental groups: GI: Negative control, GII: Positive control – Diclofenac, GIII: Problem I – *Tessaria integrifolia*-100, GIV: Problem II – *T. integrifolia*-200, GV: Problem III – *T. integrifolia*-400, GVI: Standard – Silymarin. (a). cholesterol, triglycerides, and bilirubin. (b). Aspartate aminotransferase, alanine aminotransferase, urea, and creatinine. (*): Group showed a significant difference with the GII (Diclofenac); (+): Group showed a significant difference with the GI (Control); (#): Group showed a significant difference with the GVI. Analysis of variance and Tukey’s *post hoc* test at p < 0.05.

Figure-1 shows the biochemical parameters, with AST, ALT, cholesterol, triglycerides, bilirubin, urea, and creatinine levels showing a significant increase in the diclofenac-treated group compared to the negative control group (p < 0.05). For groups administered both *T. integrifolia* extract and diclofenac, the values of the biochemical parameters were significantly lower compared to the group that received only diclofenac.

### Histopathological study

**Figure-2:**
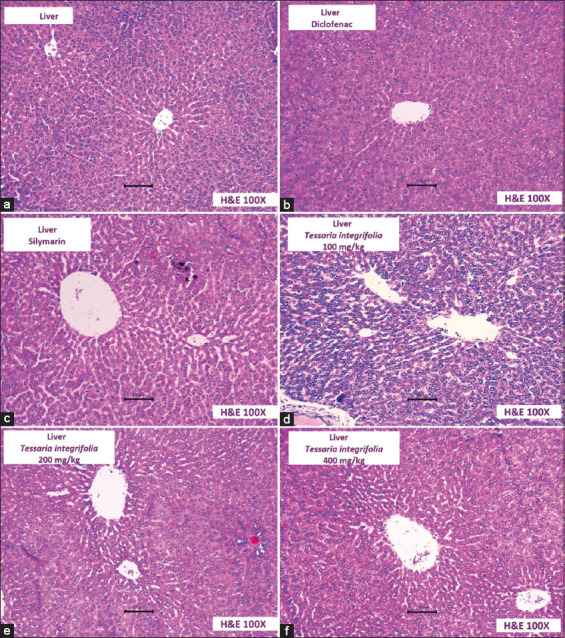
Photomicrographs of rat liver tissue (a) Control: Liver tissue showing normal architecture. (b) Diclofenac: Distorted hepatic cord arrangement around the central vein, hepatocyte necrosis, and marked sinusoidal dilatation and infiltration. (c) Silymarin: A disorder that remains in the disposition of the hepatic cords toward the central vein, and there is still necrosis of hepatocytes. (d-f) *Tessaria integrifolia*: Disposition and radial arrangement of the hepatic cords radial toward the central vein, few necrotic hepatocytes. (f) There is evidence of an improvement in liver architecture because of the dose of *T. integrifolia* in the highest dose.

Figure-2 shows the protective effect of *T. integrifolia* extracts compared to the group treated with diclofenac alone. The latter group presented liver lesions characterized by hepatocytes in a degenerative state, a distorted arrangement of hepatic cords around the central vein, necrotic zones, chromatin condensation or pyknosis, and significant sinusoidal dilation and infiltration. Thus, the hepatoprotective effect of *T. integrifolia* is evident, particularly at the highest dose.

**Figure-3:**
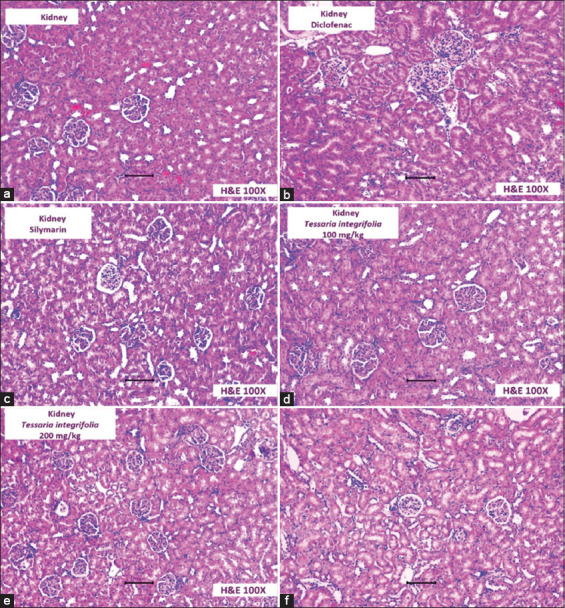
Photomicrographs of rat kidney tissue (a) Control: Kidney tissue showing normal architecture. (b) Diclofenac: Dilated renal tubules and presence necrosis of cuboidal cells of the tubular epithelium, distorted renal glomerulus. (c) Silymarin: Dilation is maintained, tubular epithelial necrosis decreases, and the presence of slight mononuclear infiltration. (d-f) *Tessaria integrifolia*: Renal tubules maintain their contour and cellular activity, little epithelial necrosis, and recovery of the renal glomerulus. (f) The histological picture is better in response to the higher dose of *T. integrifolia*.

In Figure-3, the *T. integrifolia* extracts show minor renal lesions compared to the diclofenac group, which presents dilated renal tubules, necrosis of cuboidal cells within the tubular epithelium, and distorted renal glomeruli. Thus, the nephroprotective effect of *T. integrifolia* is evident, particularly at the highest dose.

## Discussion

Liver and kidney damage are adverse effects associated with NSAIDs, with diclofenac being among the drugs carrying the highest risk of inducing such damage within this pharmacological group [20]. Metabolically, diclofenac is eliminated predominantly as a 4-hydroxylated metabolite in humans, while the acylglucuronide (GA) pathway predominates in rats [21]. Notably, GA can modify cellular proteins, covalently binding to liver proteins in rats due to the activity of multidrug resistance protein Type 2, a hepatic canalicular transporter [22].

In this study, we determined biochemical parameters. The levels of AST, ALT, cholesterol, triglycerides, bilirubin, urea, and creatinine in the diclofenac-treated group showed a significant increase compared to the negative control group (p < 0.05). Similarly, the groups that received *T. integrifolia* extracts showed significantly lower levels of these biochemical parameters than the positive control and silymarin groups, with this effect being dose-dependent (Figure-1).

Diclofenac-induced hepatotoxicity in rats is characterized by significantly decreased COX-2 and PGE2 protein expression [23–25]. Diclofenac inhibits PG production, interrupting the glomerular filtration rate and impairing renal tubular function and metabolism [26, 27]. Furthermore, diclofenac-induced nephrotoxicity is caused by renal mitochondrial damage, which produces ROS, resulting in apoptosis, DNA damage, and increased creatinine and urea levels, all of which are indicative of renal damage [26, 27]. Notably, a significant deterioration in renal function and oxidative markers has been reported following diclofenac administration in rats [28–30].

Serum markers, such as AST and ALT, are commonly measured to evaluate clinical and experimental liver damage. The specificity of ALT to the liver makes it a superior parameter for analyzing liver damage [31]. In cases of hepatotoxicity, the cellular transport of liver cells is modified, altering the plasmatic membrane, which results in the release of these liver enzymes and a subsequent increase in serum levels [32]. Elevated creatinine and urea levels are associated with the compromised integrity of the glomerular filtration rate barrier, leading to renal failure [33, 34].

At the histopathological level, the groups treated with the methanolic extract of *T. integrifolia* showed reduced liver and kidney damage compared to the diclofenac-treated group. The latter group showed liver lesions characterized by hepatocytes in a degenerative state, necrosis, and chromatin condensation or pyknosis, along with renal lesions primarily characterized by many dilated renal tubules, cuboidal epithelium cells in hydropic degeneration, necrosis, and significant distortion of the renal glomerulus (Figures-2 and 3). These changes are characteristic of diclofenac-induced liver and kidney damage [30].

Healthy kidney tissue typically contains sufficient arachidonic acid and COX. However, the conversion of arachidonic acid to PGE2 in the kidneys of diclofenac-treated rats is inhibited, impairing renal physiology by reducing renal blood flow and glomerular filtration rate, which ultimately alters electrolyte homeostasis [35, 36].

The observed protection of liver and kidney tissues stemming from *T. integrifolia* administration in this study can be attributed to the antioxidant properties of the secondary metabolites reported in the methanolic extract. Therefore, future research should focus on elucidating this protective effect [14].

## Conclusion

In this study, we evaluated biochemical parameters related to liver and kidney function in rats. We observed that the group treated with the methanolic extract of *T. integrifolia* produced significantly lower levels of cholesterol, triglycerides, bilirubin, AST, ALT, urea, and creatinine compared to the group treated with diclofenac, which induced hepatotoxicity and nephrotoxicity. This effect exhibited a dose-dependent pattern. The administration of the methanolic extract of *T. integrifolia* produced changes at the histopathological level compared to the diclofenac group, including decreased hepatocyte necrosis and reduced sinusoidal dilation and infiltration. At the renal level, administration of the *T. integrifolia* extract resulted in reduced epithelial necrosis and renal glomerulus damage. In addition, compared to the silymarin-treated group, the groups treated with *T. integrifolia* extracts showed superior hepatoprotective and nephroprotective effects, characterized by significantly lower values regarding biochemical parameters and histopathological changes.

## Authors’ Contributions

PDM: Collected the plant species and entered in the herbarium. PDM and CRS: Produced the first draft. VEV and JDR: Performed the statistical analysis and the prepared the images. AAC and CLA: Performed organ harvesting for histopathological analysis, maintained the animals during the investigation, and administered treatments. CRS, PDM, and VEV: Carried out the preparation of extract. All authors have read, reviewed, and approved the final manuscript.
